# Overexpression of serum HMGB1 and IDO in esophageal squamous cell carcinoma patients: potential clinical auxiliary diagnostic markers and immunotherapeutic targets

**DOI:** 10.3389/fonc.2024.1452282

**Published:** 2024-09-09

**Authors:** Wenxuan Cui, Yinghao Niu, Xueyuan Zhang, Beixuan Huang, Xiaoya Shang, Wei Zhao, Xi Yan, Yunqiang Mi, Ming Ma, Jinyan Zhang, Xingxiao Yang

**Affiliations:** ^1^ Clinical Laboratory, The Fourth Hospital of Hebei Medical University, Shijiazhuang, Hebei, China; ^2^ Department of Clinical Biobank, The First Hospital of Hebei Medical University, Shijiazhuang, Hebei, China; ^3^ Department of Radiotherapy, The Fourth Hospital of Hebei Medical University, Shijiazhuang, Hebei, China; ^4^ Clinical Laboratory, 984th Joint Logistic Support Force Hospital, Beijing, China; ^5^ Department of Infection Management, The Fourth Hospital of Hebei Medical University, Shijiazhuang, Hebei, China

**Keywords:** ESCC, HMGB1, IDO, lymphocyte subsets, prognosis, serum

## Abstract

**Background:**

High mobility group box 1 (HMGB1) and indoleamino-2, 3-dioxygenase (IDO) participate in the occurrence and development of esophageal squamous cell carcinoma (ESCC), regulate the tumor immune microenvironment, and are closely related to tumor growth and metastasis. However, the regulatory mechanism of serum HMGB1 and IDO has not been clarified and needs further exploration.

**Methods:**

Blood samples of 55 ESCC patients initially hospitalized in the Fourth Hospital of Hebei Medical University from August 2021 to January 2022 were selected as the ESCC group, and relevant clinical data were collected, and blood samples from 40 healthy people during the same period were selected as the control group. Serum HMGB1 and IDO levels were determined by ELISA, and lymphocyte subsets in peripheral blood of all subjects were detected by flow cytometry. The correlation between the expression levels of HMGB1 and IDO in ESCC cells was detected by Western blot.

**Results:**

Serum HMGB1 and IDO levels were significantly increased in ESCC patients, and with the progression of ESCC patients, serum HMGB1 and IDO levels were also gradually increased; serum HMGB1 was significantly correlated with IDO; serum HMGB1 and IDO combined with CEA and SCC-Ag were of high value in predicting the clinical progression of ESCC patients; the absolute counts of CD4^+^CD28^+^T cells and CD8^+^CD28^+^T cells in high HMGB1 group were significantly lower than those in low HMGB1 group, while the percentage of CD4^+^PD-1^+^T cells was significantly higher than that in low HMGB1 group; the percentage and absolute counts of CD4^+^CD28^+^T cells and the absolute counts of CD8^+^CD28^+^T cells in high IDO group were significantly lower than those in the low IDO group, while the percentage of CD8^+^PD-1^+^T cells was significantly higher than that in the low IDO group; increased serum HMGB1 and IDO expression levels were closely related to poor prognosis in ESCC patients; and HMGB1 may promote IDO expression by activating NF-κB signaling pathway.

**Conclusion:**

Serum HMGB1 and IDO have a synergistic effect, they inhibit immune function and promote tumor progression in ESCC patients, and also lead to poor prognosis.

## 1 Introduction

Esophageal carcinoma (EC) is a kind of malignant tumor of digestive tract which is highly aggressive and has a poor prognosis. Global Cancer database (GLOBOCAN) shows that in 2022, the global incidence of esophageal cancer ranked 11th and the mortality rate ranked 7th (1). China is the region with a high incidence of esophageal cancer, ranking 6th in incidence and 4th in mortality. Both the incidence and mortality of male EC are higher than those of female (2, 3). Among them, ESCC is the main pathological type of EC. At present, EC is mainly treated by surgery, combined with radiotherapy and chemotherapy, but the overall prognosis of patients is still poor (4). In recent years, the emergence of immunotherapy has made up for the deficiency of traditional therapy such as radiotherapy and chemotherapy to some extent. However, the onset of ESCC is insidious, the early diagnosis rate is low and most patients have already entered the middle or late stages when they have symptoms, which is one of the most important factors leading to the poor prognosis of ESCC (5). Therefore, it is particularly important to search for tumor markers and new immunotherapy targets, which is helpful for the auxiliary diagnosis and therapeutic effect monitoring of ESCC.

HMGB1 is a highly conserved non-histone protein widely found in eukaryotic cells. It not only participates in cell proliferation, differentiation and invasion, but also can be actively secreted by immune cells or passively secreted by apoptotic and necrotic cells to bind to a variety of receptors outside the cells, activate relevant signaling pathways, and mediate anti-tumor immune response (6, 7). Previous literature has reported that HMGB1 is highly expressed in ESCC tissues and is significantly associated with poor prognosis (8, 9).

IDO is a protein containing ferroheme, which is mainly expressed in immune cells and tumor cells, and can also be secreted into the extracellular to play a role. IDO is a key enzyme in tryptophan metabolism along kynurenine pathway, which can create a tryptophan starvation environment and accumulate metabolites to cause tumor immune tolerance and immune escape (10). At present, there are three known IDO family subtypes, namely IDO1, IDO2 and tryptophan-2, 3-dioxygenase. IDO1 is the main isotype in the IDO family, it is more widely distributed than the other two and the catalytic tryptophan activity is higher, so it has been the most widely studied (11).

In previous studies, we have demonstrated that the expression levels of HMGB1 and IDO is abnormally high in ESCC tissues, and the down-regulation of HMGB1 expression can inhibit the proliferation, migration and invasion ability of ESCC cells and promote their apoptosis, and the expression level of HMGB1 is closely related to the progression of ESCC tumors (12); At the same time, we also demonstrated that the expression level of IDO in the tumor microenvironment was positively correlated with CD3^+^CD4^+^T cells, CD3^+^CD8^+^T cells, CD3^-^CD16^+^CD56^+^NK cells, and negatively correlated with CD3^-^CD19^+^B cells and CD4^+^CD25^+^Treg cells. IDO may induce poor prognosis in ESCC patients by participating in tumor immune escape (13). Therefore, this study continued to further explore the relationship between serum HMGB1, IDO and T lymphocyte functional phenotype, as well as the correlation between serum HMGB1 and IDO expression levels and prognosis of ESCC patients, in order to provide new ideas for clinical assistant diagnosis and immunotargeted therapy of ESCC.

## 2 Materials and methods

### 2.1 Collection and statistics of clinical samples

Blood samples of 55 ESCC patients initially hospitalized in the Fourth Hospital of Hebei Medical University from August 2021 to January 2022 were collected as the ESCC group. All patients were confirmed as ESCC by imaging and pathology and had complete clinical data. None of the ESCC patients received cancer-related treatment. There were 40 males and 15 females, ranging in age from 44 to 87 years old, with a median age of 67 years old. Clinical (or pathological) staging of ESCC was performed according to the eighth edition of the American Joint Committee on Cancer (AJCC)/Union International Against Cancer (UICC) staging method. Another 40 blood samples from healthy people in the same period were selected as the control group. Among them, 27 were males and 13 were females, ranging in age from 47 to 83 years old, with a median age of 64 years old. None of the cases was associated with other serious diseases such as other tumors, serious immune diseases, serious cardiovascular diseases, and serious infectious diseases. The study was conducted in accordance with the principles of the Declaration of Helsinki, informed consent was obtained from all patients, and the Ethics Committee of the Fourth Hospital of Hebei Medical University approved the study (2021KS037).

### 2.2 The expression levels of HMGB1 and IDO in serum and cell culture supernatant were detected by enzyme-linked immunosorbent assay

Serum or cell culture supernatant of all subjects were taken out of the refrigerator at -80°C, ELISA kits for HMGB1 detection (Elabscience, Wuhan, China) and IDO detection (Elabscience, Wuhan, China) were taken out of the refrigerator at 4°C for 20 minutes in advance, and equilibrated to room temperature. The tests were performed according to the instruction of the ELISA kit. Finally, add 50uL of termination solution to each reaction hole and immediately detect the absorbance value by the enzyme-labeled instrument (PHOMO, Zhengzhou, China). Using the concentration of standard substance as the horizontal coordinate and absorbance value as the vertical coordinate, plot a standard curve in order to calculate expression levels of HMGB1 and IDO in serum and cell culture supernatant.

### 2.3 Flow cytometry was used to detect the percentage and absolute counts of T lymphocyte subsets in peripheral blood

20uL anticoagulant whole blood and 2uL monoclonal antibody CD3-ECD (Beckman Coulter, California, USA), CD45-Percp (Becton, Dickinson and Company, New Jersey, USA) CD4-PEcy7 (Becton, Dickinson and Company, New Jersey, USA), CD8-APC (Beckman Coulter, California, USA), CD28-FITC (Biolegend, California, USA), CD279 (PD-1) -PE (Biolegend, California, USA) mixed antibody were added to a 5mL flow tube. Then 150uL of red blood cell lysate (Beckman Coulter, California, USA) was added and incubated at room temperature and dark for 15 minutes. 1mL of normal saline was added and centrifuged at 2000r/min for 5 minutes. The supernatant was discarded. Each tube was added with 20uL flow-counting fluorescent microspheres (Beckman Coulter, California, USA) and measured by flow cytometry (Beckman Coulter, California, USA). Kaluza Analysis software (Beckman Coulter, California, USA) analyzed the percentage and absolute counts of CD4^+^CD28^+^T cells, CD4^+^PD-1^+^T cells, CD8^+^CD28^+^T cells and CD8^+^PD-1^+^T cells.

### 2.4 Culture and transfection of human ESCC cells

Human ESCC cell lines (KYSE30 and ECA109) were obtained from the Scientific Research Center of the Fourth Hospital of Hebei Medical University. The cells were cultured with RPMI 1640 medium containing 10% fetal bovine serum (Biological Industries, Shanghai, China) at 37°C and 5% CO_2_. KYSE30 and ECA109 cells were inoculated into a six-well plate at a concentration of 1×10^6^/L. When the bottom coverage reached 80%, Lipofectamine^®^2000 transfection reagent (Invitrogen, California, USA) and HMGB1-siRNA plasmid (GenePharma, Shanghai, China) (Sense: 5 ′ GGGAGGAGCAUAAGAAGAATT-3′; Antisense: 5 ′-UUCUUCUUAUGCUCCUCCCTT-3') and negative control siRNA (NC-siRNA) plasmid (GenePharma, Shanghai, China) (Sense: 5′-UUCUCCGAACGUGUCACGUTT-3′; Antisense: 5 ′- ACGUGACACGUUCGGAGAATT-3') was respectively prepared into a mixed suspension and transfected into KYSE30 and ECA109 cells. The final transfection concentration was 50nmol/L. The control group, si-NC group, si-HMGB1 group and si-HMGB1+PMA group were cultured with RPMI 1640 medium containing 10% fetal bovine serum 6 hours later. si-HMGB1+PMA group was added with NF-κB signaling pathway activator Phorbol 12-myristate 13-acetate (PMA) (MedChemExpress, Shanghai, China), after transfection for 24 hours.

### 2.5 Western blot analysis

After transfection for 48 hours, total protein was extracted with RIPA protein lysate (Solarbio, Beijing, China), and total protein concentration was determined with BCA protein concentration assay kit (Solarbio, Beijing, China). Added proper Loading Buffer according to the volume of superalbumin, and cook the sample in boiling water bath at 100°C for 10 minutes to get the final protein sample. The protein was transferred to PVDF membrane by 12% SDS-PAGE electrophoresis for 120 minutes. 5% skimmed milk powder was enclosed at room temperature for 2 hours and then respectively added with HMGB1 (1:10,000) (Abcam, Cambridge, UK) diluted with TBST, IDO (1:1000) (Abcam, Cambridge, UK), p65 (1:10,000) (Abcam, Cambridge, UK) and GAPDH (1:500) (Abbkine, New Jersey, USA) rabbit primary antibody was incubated at 4°C for at least 16 hours, and then TBST-diluted rabbit secondary antibody (1:10,000) (Tiandeyue Biotechnology Co., Beijing, China) and incubated for 1 hour. ECL chemiluminescence solution (Solarbio, Beijing, China) was exposed. The Image J software detected the gray values of HMGB1, IDO, p65 and GAPDH strips. The experiment was repeated independently for 3 times.

### 2.6 Statistical analysis

SPSS 26.0 was used for statistical analysis, and GraphPad Prism 8.4.0 software was used for mapping. The normal distribution of measurement data was expressed by mean ± standard ( $\bar{x}\;\pm \;s$ ) deviation, the skewed distribution was expressed by the median (*M*) [interquartile (*P*_25_, *P*_75_)]. Mann-Whitney *U* test was used to compare the serum HMGB1 and IDO levels between the control group and ESCC group and ESCC patients with different clinical stages, and analyze the correlation between different levels of serum HMGB1, IDO and T lymphocyte subsets. Spearman correlation test analyzed the levels of serum HMGB1 and IDO in ESCC patients. Receiver operating characteristic curve (ROC) was plotted to analyze the auxiliary diagnosis value of HMGB1 and IDO in both the normal people and ESCC patients, and the predictive value of serum tumor markers (CEA and SCC-Ag) and HMGB1, IDO of the clinical progression in ESCC patients. Delong test was used to analyze the significance of area under the ROC curves. The correlation between HMGB1 and IDO and the clinicopathological features of ESCC patients was analyzed by *χ^2^* test. Kaplan-Meier curves of ESCC patients with different levels of serum HMGB1, IDO were analyzed by Log-rank test. Multivariate Cox regression model was constructed to analyze the effects of serum HMGB1, IDO, CEA and SCC-Ag on the survival status of ESCC patients. One-way ANOVA compared the mean difference between multiple sets of data for ESCC cells, The minimum significance difference test (LSD) was used to analyze the multi-group comparison. *P*<0.05 was considered as statistically significant difference.

## 3 Results

### 3.1 Expression levels of serum HMGB1 and IDO between normal people and ESCC patients

**Figure 1:**
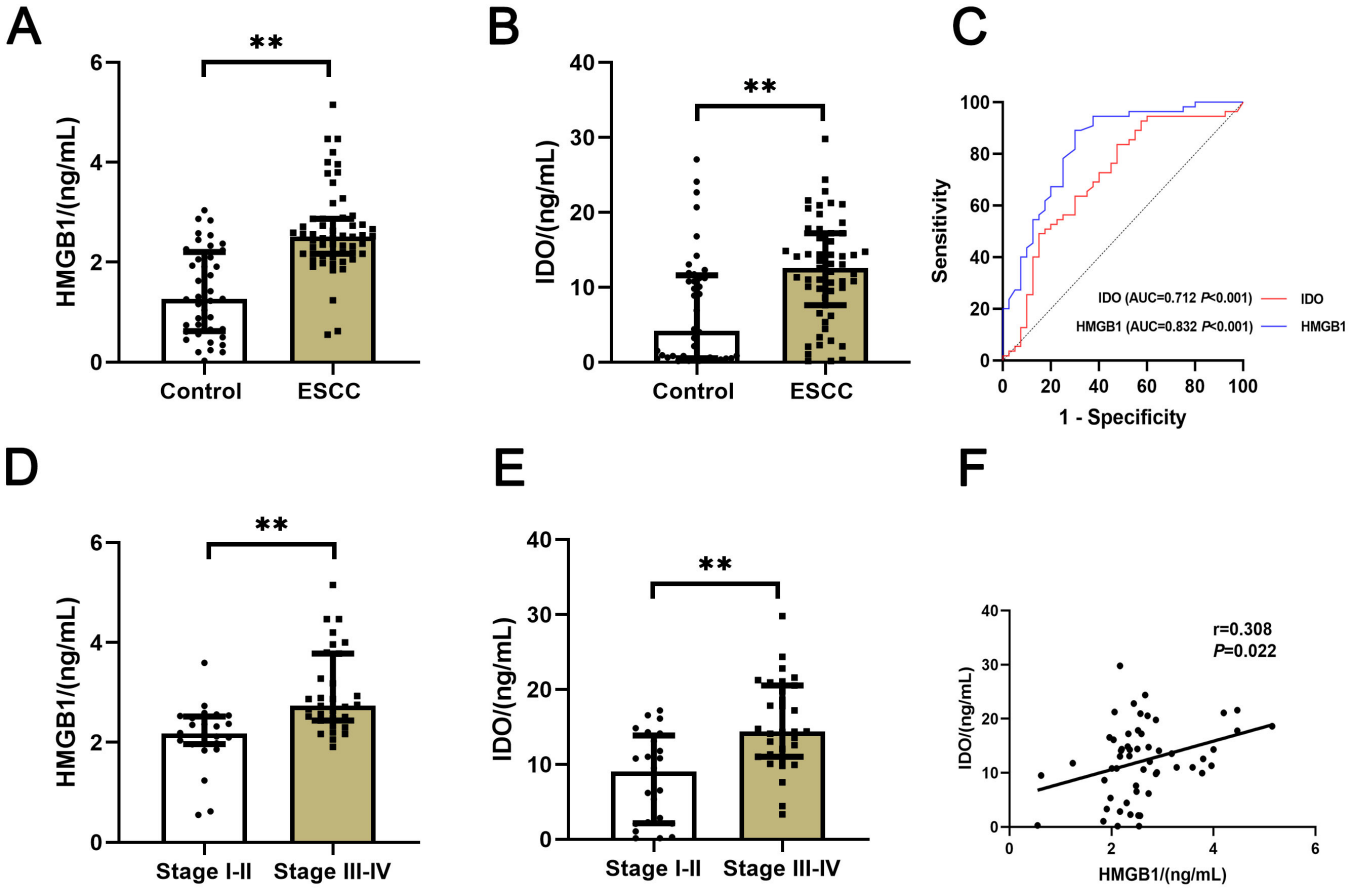
Comparison of serum HMGB1 and IDO expression levels in different groups. **(A)** Comparison of serum HMGB1 expression levels between control group and ESCC group. **(B)** Comparison of serum IDO expression levels between control group and ESCC group. **(C)** ROC curves of the auxiliary diagnosis of serum HMGB1 and IDO in both the normal people and ESCC patients. **(D)** Comparison of serum HMGB1 expression levels in ESCC patients with clinical stages I-II and III-IV. **(E)** Comparison of serum IDO expression levels in ESCC patients with clinical stages I-II and III-IV. **(F)** Correlation analysis of serum HMGB1 and IDO. **P<0.001.

The expression levels of serum HMGB1 and IDO in all groups were compared. The results showed that the expression levels of serum HMGB1 and IDO in ESCC group were significantly higher than those in control group (*P*<0.001) (**Figures 1A, B**), They had good value of auxiliary diagnosis in both the healthy people and ESCC patients (*P*<0.001) (**Figure 1C**). And the expression levels of serum HMGB1 and IDO in ESCC patients in clinical stage III-IV were significantly higher than those in stage I-II (*P*<0.001) (**Figures 1D, E**). Meanwhile, the expression levels of serum HMGB1 and IDO in ESCC patients were closely correlated (r=0.308, *P*=0.022) (**Figure 1F**). These results suggest that serum HMGB1 and IDO levels in ESCC patients are significantly higher than those in healthy people, and with the progression of the disease in ESCC patients, serum HMGB1 and IDO levels are gradually increasing, which may have a synergistic effect in the progression of the disease in ESCC patients.

### 3.2 Predictive value of serum HMGB1, IDO and traditional ESCC serum tumor markers for clinical progression in ESCC patients

**Figure 2:**
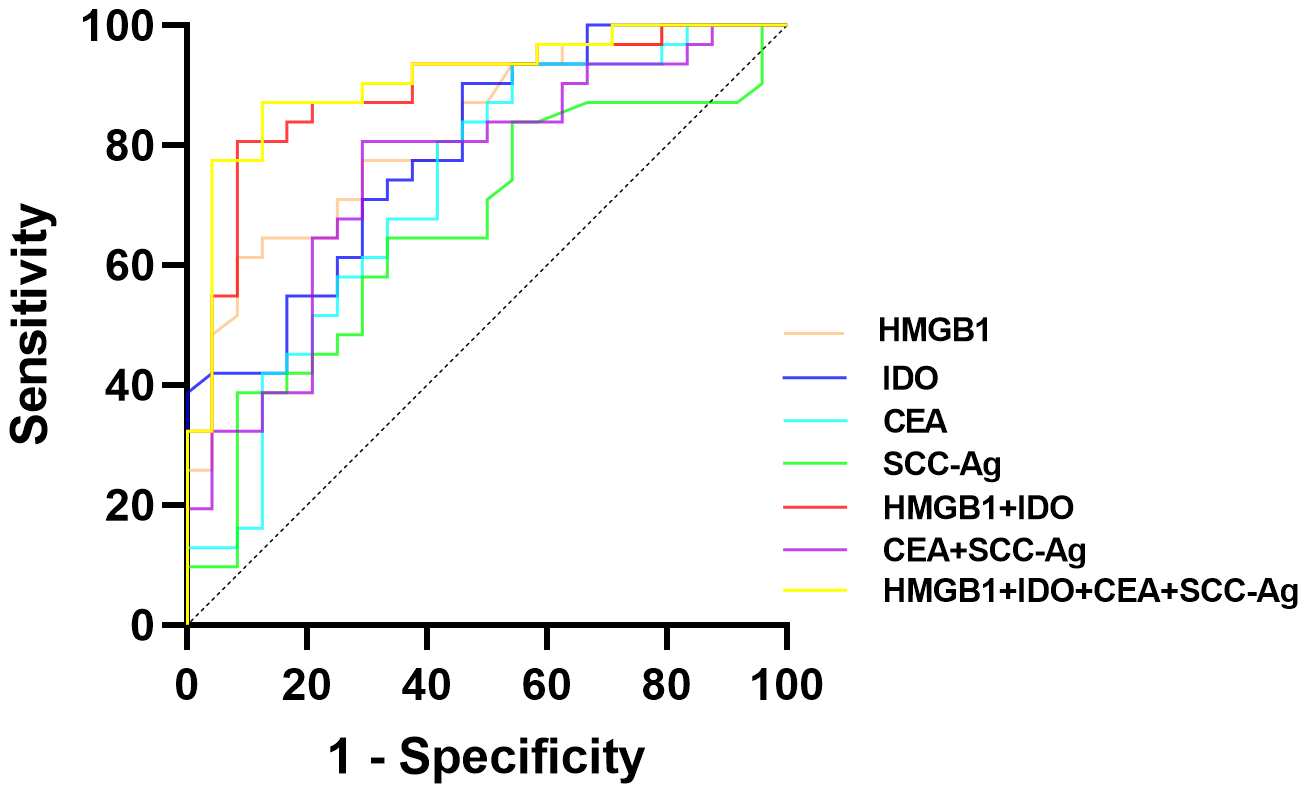
ROC curves of the predictive value of serum HMGB1, IDO, CEA and SCC-Ag in the clinical progression of ESCC patients.

The predictive value of serum HMGB1, IDO and ESCC serum tumor markers (CEA and SCC-Ag) on the clinical progression of ESCC was analyzed. ROC curves results showed that serum HMGB1, IDO, CEA and SCC-Ag all had predictive ability for clinical progression of ESCC, among which the predictive value of combined detection of serum HMGB1 and IDO (AUC=0.888, *P*<0.001) was significantly higher than that of CEA and SCC-Ag (AUC=0.754, *P*=0.001). The four-part combined test has the highest predictive value for predicting clinical progress in ESCC patients and the largest area under the curve (AUC=0.906, *P*<0.001) (**Table 1**; **Figure 2**). These results indicate that serum HMGB, IDO, CEA and SCC-Ag may have similar roles in predicting the progression of ESCC, the combined detection of serum HMGB1 and IDO has a higher predictive value than the combined detection of CEA and SCC-Ag, and the combined detection of the four is more valuable in predicting the progression of ESCC.

**Table 1:** ROC curves analysis of serum HMGB1, IDO, CEA and SCC-Ag on clinical progression of ESCC patients.

Characteristics	AUC	95% CI	*P* value	Sensitivity (%)	Specificity (%)	Cut off
HMGB1 (ng/mL)	0.819	0.710, 0.929	<0.001	61.3	91.7	2.61
IDO (ng/mL)	0.791	0.674, 0.908	<0.001	90.3	54.2	9.66
CEA (ng/mL)	0.727	0.589, 0.865	0.004	93.5	45.8	1.46
SCC-Ag (ng/mL)	0.661	0.515, 0.807	0.042	64.5	66.7	1.48
HMGB1+IDO	0.888	0.799, 0.978	<0.001	80.6	91.7	—
CEA+SCC-Ag	0.754	0.624, 0.884	0.001	80.6	70.8	—
HMGB1+IDO+CEA+SCC-Ag	0.906	0.824, 0.988	<0.001	77.4	95.8	—

### 3.3 Correlation between serum HMGB1, IDO expression levels and clinicopathological features of ESCC patients

The relationships between serum HMGB1, IDO and clinicopathological parameters of ESCC patients were analyzed. According to the cut-off values of serum HMGB1 and IDO (2.61ng/mL and 9.66ng/mL) obtained by ROC curve analysis, ESCC patients with different expression levels of HMGB1 were divided into low group (<2.61ng/mL) and high group (≥2.61ng/mL). And the different expression levels of IDO were divided into low group (< 9.66ng/mL) and high group (≥9.66ng/mL). Serum HMGB1 and IDO expression levels were significantly correlated with lymph node metastasis and clinical stage (*P*<0.001), but there was no significant correlation with age, sex and depth of tumor invasion (*P*>0.05) (**Tables 2**, **3**). These findings suggest that elevated levels of HMGB1 or IDO in ESCC patients are associated with an increased likelihood of lymph node metastasis and a more advanced clinical stage.

**Table 2:** Correlation between serum HMGB1 expression level and clinicopathological features of ESCC patients.

Characteristics		*n*	Low HMGB1 (%)	High HMGB1 (%)	*χ^2^* value	*P* value
Age	<68	26	15 (44.1)	11 (52.4)	0.356	0.551
	≥68	29	19 (55.9)	10 (47.6)		
Sex	Female	15	11 (32.4)	4 (19.0)	0.585	0.444
	Male	40	23 (67.6)	17 (81.0)		
Tumor invasion depth	<4.5cm	26	15 (44.1)	11 (52.4)	0.356	0.551
	≥4.5cm	29	19 (55.9)	10 (47.6)		
Lymph node metastasis	Negative	23	22 (64.7)	1 (4.8)	16.788	<0.001
	Positive	32	12 (35.3)	20 (95.2)		
Clinical stage	I~II	24	22 (64.7)	2 (9.5)	13.907	<0.001
	III~IV	31	12 (35.3)	19 (90.5)		

**Table 3:** Correlation between serum IDO expression level and clinicopathological features of ESCC patients.

Characteristics		*n*	Low IDO (%)	High IDO (%)	*χ^2^* value	*P* value
Age	<68	26	7 (43.8)	19 (48.7)	0.112	0.737
	≥68	29	9 (56.3)	20 (51.3)		
Sex	Female	15	6 (37.5)	9 (23.1)	1.190	0.275
	Male	40	10 (62.5)	30 (76.9)		
Tumor invasion depth	<4.5cm	26	10 (62.5)	16 (41.0)	2.099	0.141
	≥4.5cm	29	6 (37.5)	23 (59.0)		
Lymph node metastasis	Negative	23	13 (81.3)	10 (25.6)	12.225	<0.001
	Positive	32	3 (18.8)	29 (74.4)		
Clinical stage	I~II	24	13 (81.3)	11 (28.2)	10.912	<0.001
	III~IV	31	3 (18.8)	28 (71.8)		

### 3.4 Correlation between serum HMGB1, IDO expression levels and T lymphocytes in ESCC patients

**Figure 3:**
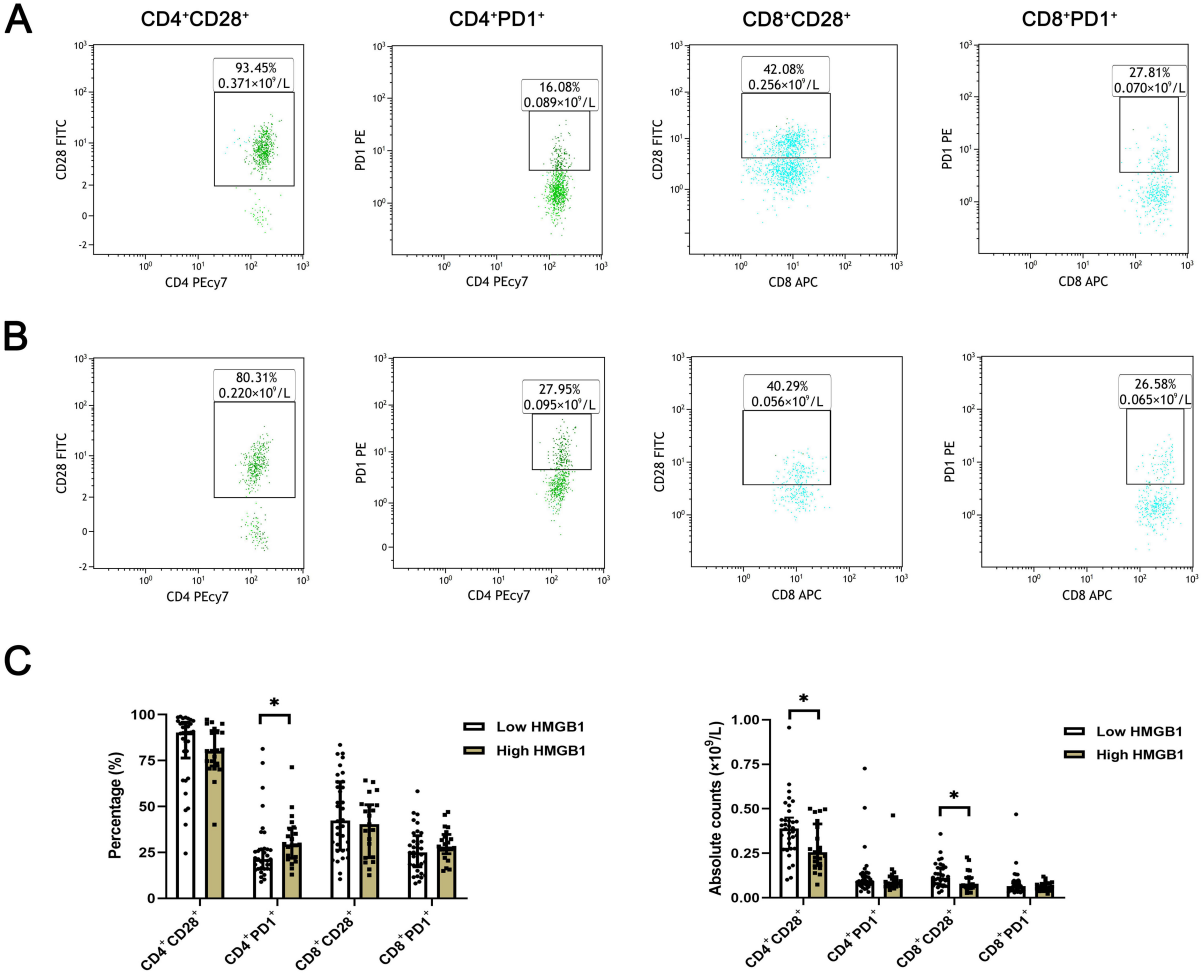
Representative flow diagram of peripheral blood T lymphocyte subsets in different levels of HMGB1. **(A)** Representative flow diagram of peripheral blood T lymphocyte subsets in low HMGB1 group. **(B)** Representative flow diagram of peripheral blood T lymphocyte subsets in high HMGB1 group. **(C)** Comparison of percentage and absolute counts of T lymphocyte subsets in high HMGB1group and low HMGB1 group. *P<0.05.

**Figure 4:**
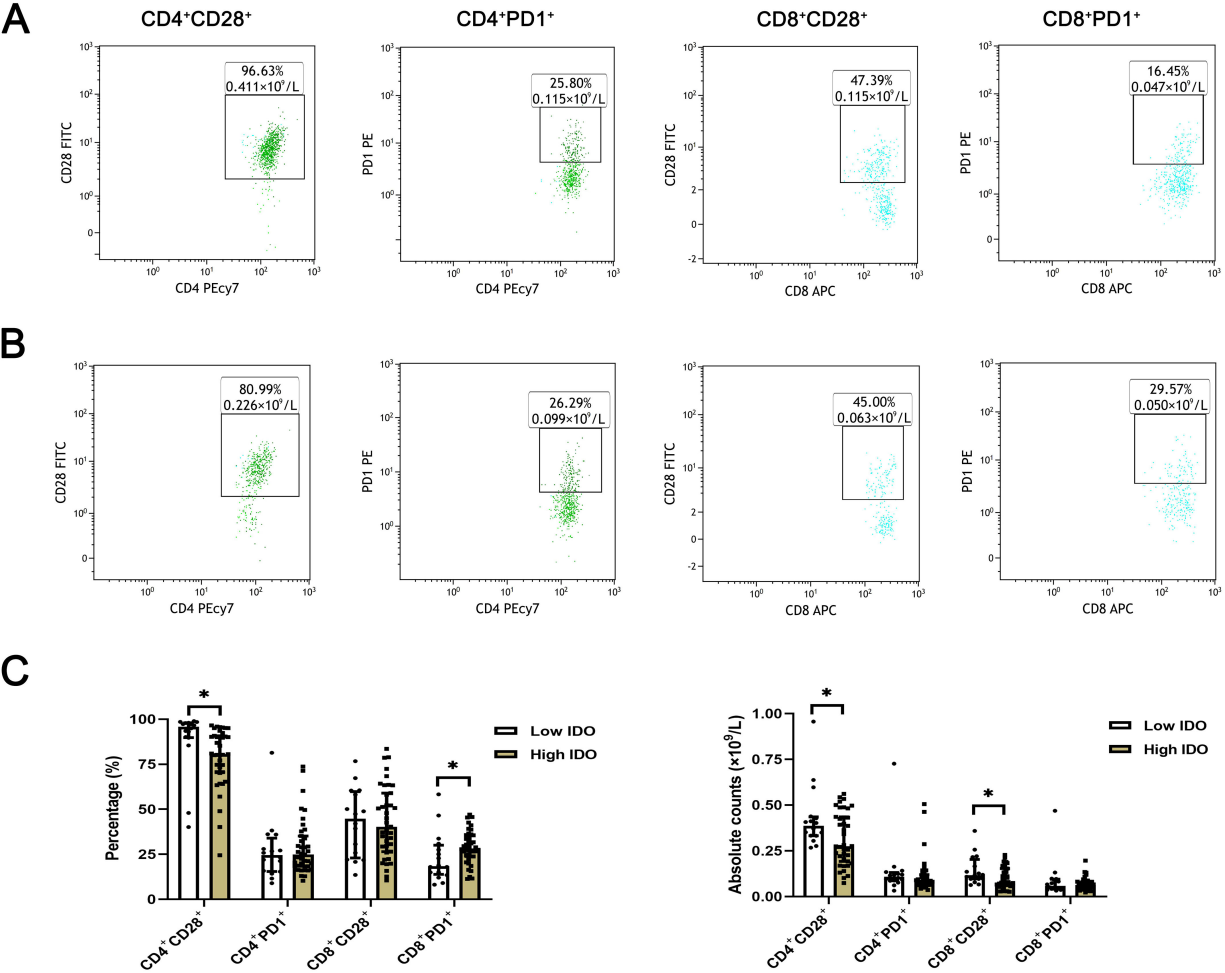
Representative flow maps of peripheral blood T lymphocyte subsets in different levels of IDO. **(A)** Representative flow diagram of peripheral blood T lymphocyte subsets in low IDO group. **(B)** Representative flow diagram of peripheral blood T lymphocyte subsets in the high IDO group. **(C)** Comparison of percentage and absolute counts of T lymphocyte subsets in high IDO group and low IDO group. *P<0.05.

The expression levels of T lymphocyte subsets in whole blood of ESCC patients were detected by flow cytometry. The absolute counts of CD4^+^CD28^+^T cells (*P*=0.013) and CD8^+^CD28^+^T cells (*P*=0.018) in the high HMGB1 group were significantly lower than those in the low HMGB1 group, while the percentage of CD4^+^PD-1^+^T cells (*P*=0.018) was significantly higher than that in the low HMGB1 group (**Table 4**; **Figure 3**); the percentage and absolute value counts of CD4^+^CD28^+^T cells (*P*=0.002, *P*=0.047) and the absolute value counts of CD8^+^CD28^+^T cells (*P*=0.029) in the high IDO group were significantly lower than those in the low IDO group, while the percentage of CD8^+^PD-1^+^T cells (*P*=0.030) was significantly higher than that in the low IDO group (**Table 5**; **Figure 4**). These results suggest that serum HMGB1 and IDO are closely related to the functional phenotype of lymphocytes in ESCC patients, and may be involved in the immune tolerance and immune escape of tumors.

**Table 4:** Correlation analysis of serum HMGB1 and T lymphocyte subsets.

Characteristics	Low HMGB1	High HMGB1	*Z* value	*P* value
CD4^+^CD28^+^ (%)	90.22 (76.34, 95.88)	80.31 (72.02, 91.37)	-1.438	0.150
CD4^+^CD28^+^ (×10^9^/L)	0.39 (0.28, 0.45)	0.26 (0.18, 0.41)	-2.477	0.013
CD4^+^PD-1^+^ (%)	21.34 (16.08, 26.81)	29.30 (21.90, 38.20)	-2.373	0.018
CD4^+^PD-1^+^ (×10^9^/L)	0.09 (0.07, 0.14)	0.09 (0.06, 0.12)	-0.563	0.573
CD8^+^CD28^+^ (%)	42.34 (26.33, 63.44)	40.29 (22.20, 51.00)	-1.057	0.291
CD8^+^CD28^+^ (×10^9^/L)	0.11 (0.08, 0.17)	0.07 (0.05, 0.11)	-2.365	0.018
CD8^+^PD-1^+^ (%)	24.98 (17.21, 34.16)	28.18 (24.28, 34.82)	-1.291	0.197
CD8^+^PD-1^+^ (×10^9^/L)	0.06 (0.04, 0.09)	0.06 (0.04, 0.08)	-0.277	0.782

**Table 5:** Correlation analysis of serum IDO and T lymphocyte subsets.

Characteristics	Low IDO	High IDO	*Z* value	*P* value
CD4^+^CD28^+^ (%)	95.79 (89.80, 98.02)	80.66 (70.63, 90.82)	-3.141	0.002
CD4^+^CD28^+^ (×10^9^/L)	0.39 (0.33, 0.44)	0.28 (0.19, 0.44)	-1.983	0.047
CD4^+^PD-1^+^ (%)	24.56 (15.49, 33.95)	24.88 (18.35, 35.12)	-0.574	0.566
CD4^+^PD-1^+^ (×10^9^/L)	0.11 (0.09, 0.13)	0.09 (0.06, 0.12)	-1.344	0.179
CD8^+^CD28^+^ (%)	44.74 (22.96, 59.83)	40.29 (26.46, 58.92)	-0.093	0.926
CD8^+^CD28^+^ (×10^9^/L)	0.12 (0.10, 0.20)	0.08 (0.06, 0.15)	-2.187	0.029
CD8^+^PD-1^+^ (%)	18.40 (13.84, 30.04)	28.18 (23.81, 35.62)	-2.168	0.030
CD8^+^PD-1^+^ (×10^9^/L)	0.06 (0.04, 0.09)	0.06 (0.04, 0.08)	-0.213	0.831

### 3.5 Correlation between serum HMGB1, IDO expression levels and prognosis of ESCC patients

**Figure 5:**
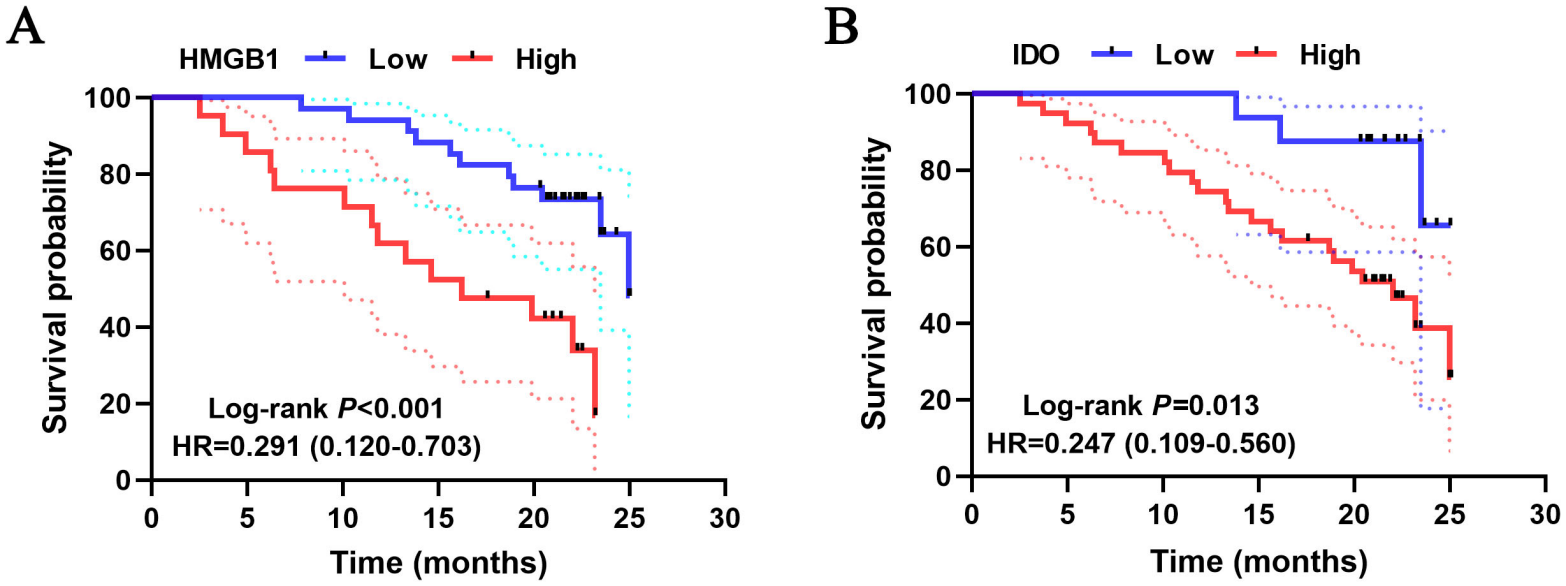
Survival analysis curves of the different levels of serum HMGB1 and IDO in ESCC patients.

Kaplan-Meier curves results showed that the survival rate of ESCC patients in the high HMGB1 group was significantly lower than that in the low HMGB1 group (95%CI=0.120-0.703, *P*<0.001) (**Figure 5A**), and the survival rate of ESCC patients in the high IDO group was significantly lower than that in the low IDO group (95%CI=0.109-0.560, *P*=0.013) (**Figure 5B**). Multivariate Cox regression results showed that HMGB1 was an independent risk factor for prognosis in ESCC patients (*P*=0.035), however, IDO is not an independent risk factor for the prognosis of ESCC patients (*P*>0.05) (**Table 6**). The results suggest that high levels of serum HMGB1 may lead to poor prognosis in ESCC patients, HMGB1 has higher value than IDO in predicting the prognosis of ESCC patients. HMGB1 is a more important marker for the prognosis of ESCC than IDO.

**Table 6:** Cox regression analysis of serum HMGB1 and IDO at different levels in ESCC patients.

Characteristics	Univariate analysis		Multivariate analysis	
	Hazard ratio (95% CI)	*P* value	Hazard ratio (95% CI)	*P* value
HMGB1 (≥2.61 ng/mL)	3.807 (1.639, 8.843)	0.002	2.658 (1.073, 6.579)	0.035
IDO (≥9.66 ng/mL)	4.100 (1.223, 13.747)	0.022	2.359 (0.633, 8.801)	0.201
CEA (≥1.46 ng/mL)	3.465 (1.028, 11.674)	0.045	2.728 (0.795, 9.366)	0.111
SCC-Ag (≥1.48 ng/mL)	0.592 (0.252, 1.390)	0.228		

### 3.6 HMGB1 promotes IDO expression level in ESCC cells through NF-κB signaling pathway

**Figure 6:**
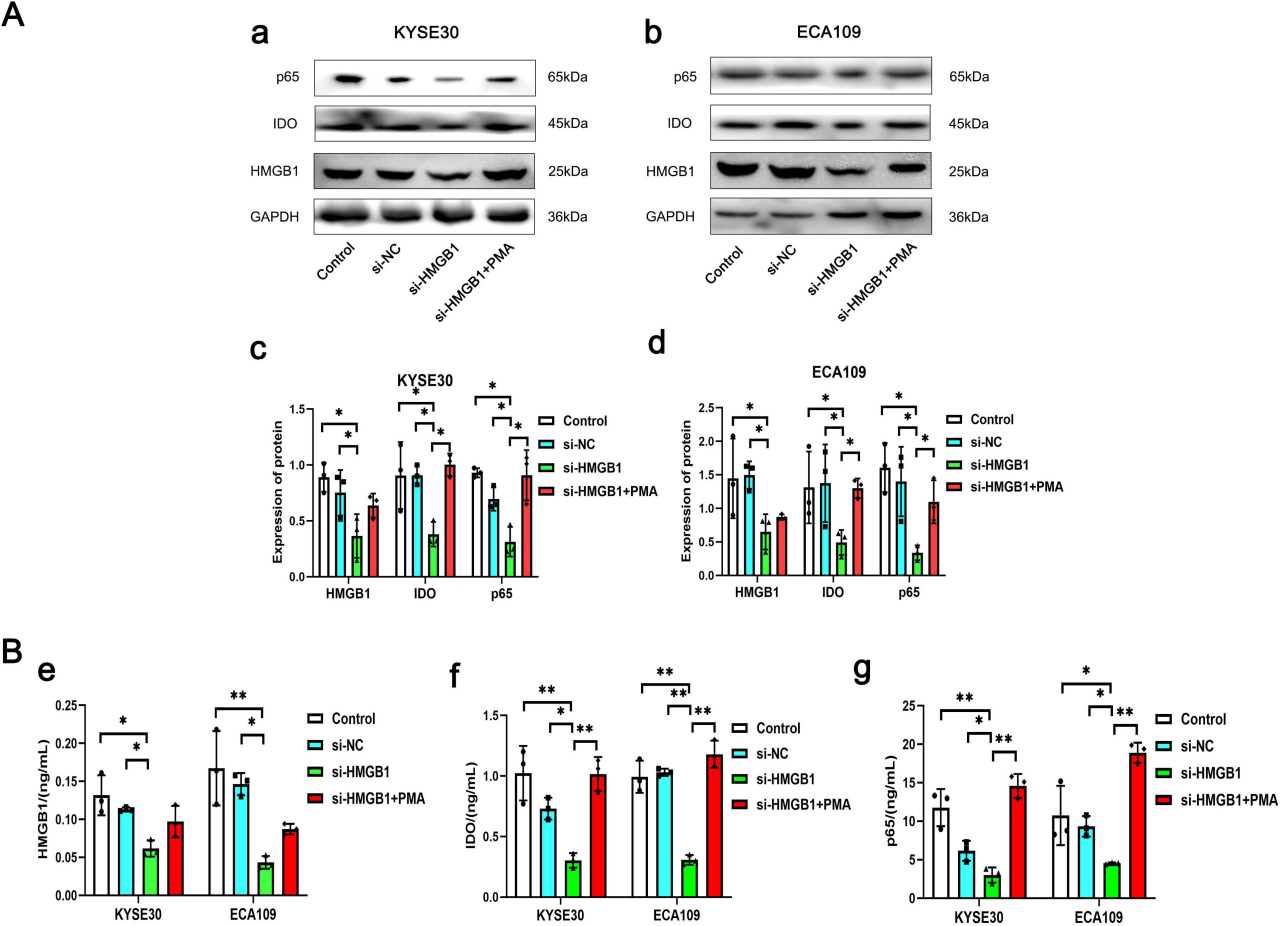
The expression levels of HMGB1, IDO and p65 in different groups of KYSE30 and ECA109 cells. **(A)** Comparison of the expression levels of HMGB1, IDO and p65 in control group, si-NC group, si-HMGB1 group and si-HMGB1+PMA group of KYSE30 (a, c) and ECA109 cells (b, d). **(B)** Comparison of HMGB1 (e), IDO (f) and p65 (g) expression levels in control group, si-NC group, si-HMGB1 group and si-HMGB1+PMA group of KYSE30 and ECA109 cell culture supernatant. *P<0.05, **P<0.001.

Western blot results showed that after HMGB1 gene expression was knocked out in KYSE30 and ECA109 cells, the expression level of IDO was also significantly decreased (*P*=0.006, *P*=0.030), and after the addition of NF-κB signaling pathway activator (PMA), the level of IDO was significantly increased (*P*=0.002, *P*=0.043), while the level of HMGB1 was only slightly increased (*P*>0.05) (**Figure 6A**). At the same time, the results of cell culture supernatant were consistent with those of Western blot. In the cell culture supernatant of KYSE30 and ECA109 cells, the expression level of IDO was also significantly decreased (*P*=0.006, *P*<0.001), and after the addition of NF-κB signaling pathway activator (PMA), the level of IDO was significantly increased (*P*<0.001, *P*<0.001) (**Figure 6B**). These indicate that HMGB1 in ESCC cells may promote the expression level of IDO by activating the NF-κB signaling pathway, and at the same time, the levels of HMGB1 and IDO in the cell culture supernatant also changed.

## 4 Discussion

HMGB1 is widely present in eukaryotic cells. When the body is stimulated by the outside world, HMGB1 can be passively secreted into the extracellular by damaged and necrotic cells or actively secreted by immune cells to participate in the occurrence and development of tumors, which is closely related to the clinical progress and prognosis of patients (14, 15). It has been reported that HMGB1 can be secreted extracellular by ESCC cells in the form of exosomes to promote the proliferation of tumor cells (16). IDO can be used as an exosomal tumor immune protein to predict the clinical progression of patients with non-small cell lung cancer (17). But it is less studied in ESCC.

According to the statistical results of this study, the expression levels of serum HMGB1 and IDO in ESCC patients were significantly higher than those in healthy people, they also had good value of auxiliary diagnosis in both the healthy people and ESCC patients. And with the aggravation of the disease, the expression levels of serum HMGB1 and IDO in ESCC patients were also significantly increased, and the expression levels of the two were closely related in ESCC patients. The analysis of clinical data related to ESCC patients showed that serum HMGB1 and IDO were closely related to lymph node metastasis and clinical stage.

At present, there are not many serum tumor markers that have been mature for clinical auxiliary diagnosis and prognosis assessment of ESCC. Therefore, it has great clinical significance to search for serum biomarkers with higher stability, sensitivity and specificity. In this study, the abilities of serum HMGB1, IDO, CEA and SCC-Ag expression levels to predict clinical progression in ESCC patients were analyzed. The results showed that HMGB1 and IDO were similar to CEA and SCC-Ag, both of which could better reflect the progression of ESCC tumors. Moreover, ROC curve analysis results suggested that the accuracies of serum HMGB1 and IDO were higher than CEA and SCC-Ag, whether detected alone or in combination. It shows that serum HMGB1 and IDO have high predictive value for clinical progression of ESCC patients. In addition, the results of this study also showed that the combined detection of serum HMGB1, IDO and SCC-Ag had better diagnostic efficacy, and relatively high sensitivity and specificity. The above results further reflect the potential of serum HMGB1 and IDO as tumor markers of ESCC.

Next, we detected the expression levels of peripheral blood CD4^+^CD28^+^T cells, CD4^+^PD-1^+^T cells, CD8^+^CD28^+^T cells and CD8^+^PD-1^+^T cells by flow cytometry, and explored the correlation between serum HMGB1, IDO and surface receptors CD28 and PD-1 of CD4^+^T cells and CD8^+^T cells. Both CD28 and PD-1 are members of the B7-CD28 superfamily (18). The B7-CD28 superfamily is involved in the second signal of T cell activation and is an important factor affecting T cell response. The co-stimulatory molecules B7.1/B7.2 (also known as CD80 and CD86) in the B7 family, when B7.1 is stimulated by antigen, B7.1 and B7.2 are highly expressed in antigen presenting cells (APCs), and interact with activated phenotype CD28 to induce T cells to produce IL-2, thereby inducing T cell proliferation (19). PD-1/PD-L1 is a co-inhibitory molecule in the B7 family. During the activation of T cells, PD-L1 binds to the inhibitory phenotype PD-1 to promote the production of immunosuppressive cytokine IL-10, thereby inhibiting the immune response of T cells (19).

Previous literature has suggested that serum HMGB1 was closely related to the level of peripheral blood T lymphocyte subsets, and has a dual regulatory effect on the body’s immune function (20, 21). On the one hand, HMGB1, as a damage-associated molecular pattern molecule (DAMP), can induce the maturation of dendritic cells (DCs), thereby promoting the generation and activation of CD8^+^T cells and promoting anti-tumor immune response (22, 23). On the other hand, HMGB1 can cause the body’s immune function to be inhibited (24). Our results showed that with the increase of serum HMGB1 expression level, the absolute counts of CD4^+^CD28^+^T cells and CD8^+^CD28^+^T cells decreased significantly, while the percentage of CD4^+^PD-1^+^T cells increased significantly. Wang established a mouse model of sepsis and found that plasma HMGB1 level was increased in mice with sepsis, while the expression of CD28 on the surface of CD4^+^T cells was inhibited, and the expression of PD-1 was increased (25). This is consistent with the results of our study. It suggests that high level of HMGB1 can inhibit the function of T cells and induce immune escape. In tumor immunotherapy, blocking the expression of extracellular HMGB1 can improve the efficacy of anti-PD-1 tumor immunotherapy (26). The release of HMGB1 can further trigger the expression of CD274 (PD-L1) in tumor cells and inhibit T cell immune function (27). The findings suggest a close association between HMGB1 and the immune checkpoint PD-1/PD-L1, indicating that serum HMGB1 may exert inhibitory effects on T cell proliferation and activation by modulating the expression of CD28 and PD-1 on T cell surfaces, which plays a pivotal role in tumor immune evasion.

IDO plays a dual role in tumor immune response (28, 29). Among them, the immunosuppressive effect of IDO has been relatively more widely studied. The results of this study revealed that with the increase of serum IDO expression level, the percentage and absolute counts of CD4^+^CD28^+^T cells and the absolute counts of CD8^+^CD28^+^T cells were significantly decreased, while the percentage of CD8^+^PD-1^+^T cells was significantly increased. This result may be due to the stronger binding of cytotoxic T-cell-associated protein-4 (CTLA-4) to CD80/CD86 than CD28, while activating IDO, which inhibits T cell activity through tryptophan depletion or local accumulation of inhibitory metabolites (30). Liu found that decreased tryptophan expression and increased IDO1 expression were associated with increased PD-1 expression on the surface of cytotoxic T cells (31). This is consistent with the results of this study. The combined application of IDO as an immunosuppressant and PD-1/PD-L1 immunosuppressant provides a new idea for clinical tumor immunotherapy (32). These results suggest that IDO may inhibit the function of T cells and promote tumor immune escape by affecting the functional phenotype of T cells.

At the same time, we found that overexpression of serum HMGB1 was significantly associated with shorter survival in ESCC patients and was an independent risk factor for prognosis in ESCC patients. Li showed that the expression of HMGB1 was strongly correlated with the clinicopathological features of ESCC patients, and high expression of HMGB1 could shorten the survival of ESCC patients (15). High expression of serum IDO is closely associated with poor prognosis in ESCC patients, which is consistent with our previous findings (12). However, multivariate Cox regression results showed that IDO had no significant correlation with the prognosis of ESCC patients, suggesting that serum HMGB1 was more valuable than IDO in predicting the prognosis of ESCC patients. Although serum IDO cannot be used as an independent risk factor for the prognosis of ESCC patients, its application to Cox proportional risk model can provide certain theoretical basis, indicating that elevated serum IDO level is a risk factor for poor prognosis of ESCC patients.

In the previous part of our study, the expression levels of serum HMGB1 and IDO were significantly positively correlated. Up to now, no literature has explored the direct regulatory relationship between HMGB1 and IDO in tumor tissues, and only previous studies have suggested that there may be a related regulatory mechanism between them. Overexpression of HMGB1 in tumor tissue can promote the proliferation and invasion of Hela cells through NF-κB signaling pathway (33). HIV-1 Tat protein can also promote the proliferation and invasion of Hela cells by enhancing the expression of IDO through NF-κB pathway (34). HMGB1 in the tumor microenvironment activates MDSCs via the NF-κB signaling pathway (35), and MDSCs produce high levels of immunosuppressive molecules (such as IDO) that inhibit T cell function (36). Therefore, we can preliminarily speculate that HMGB1 may promote the expression of IDO through NF-κB signaling pathway. According to the above literature reports, we constructed the vitro ESCC tumor models and designed relevant experiments, the results showed that after knocking down the expression of HMGB1 and adding PMA, the expression level of IDO was significantly changed, while HMGB1 level increased only slightly, which may be due to the activation of NF-κB signaling pathway to induce the expression of HMGB1 receptor, and then promote the expression of HMGB1 (37). The results of cell culture supernatant were consistent with this. These results show that HMGB1 and IDO are closely related to NF-κB signaling pathway, suggesting that serum HMGB1 and IDO may be due to the increased expression of intracellular HMGB1 in ESCC, which may activate NF-κB signaling to promote the expression of IDO and release HMGB1 and IDO to the outside of cells.

The disease of cancer patients is complex and diverse, and single therapy has certain limitations, while the combination of multiple methods to achieve complementary advantages can improve the clinical treatment effect. Kang found that the combination of the EGFR/HER2 inhibitors gefitinib and lapatinib with the IGF-1R inhibitor Lincitinib overcomes monotherapy resistance and enhances the antitumor efficacy of ESCC (38). Based on the potential association between HMGB1 and IDO, dual-target combination therapy with HMGB1 and IDO inhibitors may have better efficacy than tumor immunotherapy by inhibiting the activity of HMGB1 or IDO alone in the future.

In summary, serum HMGB1 and IDO are abnormally high expressed in ESCC patients, and HMGB1 may promote the expression of IDO through NF-κB signaling pathway, which may regulate the functional phenotype of T lymphocytes, inhibit the immune function of the body, promote tumor progression in ESCC patients and cause poor prognosis. However, our study also has some limitations, such as collecting only cases from 2021 to 2022 and being unable to assess the association of HMGB1 and IDO with five-year survival in ESCC patients. The correlation model of combined detection of tumor markers has not been established to evaluate its value in differential diagnosis and prediction of clinical progression in different tumor types, so it is not clear whether the combined detection of serum HMGB1 and IDO has high tissue specificity in differential diagnosis and prediction of clinical progression of ESCC. It was not investigated whether the expression of HMGB1 and IDO could be interfered at the animal levels to achieve a better treatment of ESCC. Next, we will expand the sample size and construct a tumor immune microenvironment model to further explore the role and related mechanisms of HMGB1 and IDO in tumor immunity.

## Data Availability

The original contributions presented in the study are included in the article/supplementary material. Further inquiries can be directed to the corresponding authors.
